# WHO PUBLISHES LIST OF BACTERIA FOR WHICH NEW ANTIBIOTICS ARE URGENTLY NEEDED

**Published:** 2017-04

**27 FEBRUARY 2017 | GENEVA** - WHO today published its first ever list of antibiotic-resistant “priority pathogens” – a catalogue of 12 families of bacteria that pose the greatest threat to human health.

The list was drawn up in a bid to guide and promote research and development (R&D) of new antibiotics, as part of WHO’s efforts to address growing global resistance to antimicrobial medicines.

The list highlights in particular the threat of gram-negative bacteria that are resistant to multiple antibiotics. These bacteria have built-in abilities to find new ways to resist treatment and can pass along genetic material that allows other bacteria to become drug-resistant as well.

“This list is a new tool to ensure R&D responds to urgent public health needs,” says Dr Marie-Paule Kieny, WHO’s Assistant Director-General for Health Systems and Innovation. “Antibiotic resistance is growing, and we are fast running out of treatment options. If we leave it to market forces alone, the new antibiotics we most urgently need are not going to be developed in time.”

The WHO list is divided into three categories according to the urgency of need for new antibiotics: critical, high and medium priority.

The most critical group of all includes multidrug resistant bacteria that pose a particular threat in hospitals, nursing homes, and among patients whose care requires devices such as ventilators and blood catheters. They include Acinetobacter, Pseudomonas and various Enterobacteriaceae (including Klebsiella, E. coli, Serratia, and Proteus). They can cause severe and often deadly infections such as bloodstream infections and pneumonia.

These bacteria have become resistant to a large number of antibiotics, including carbapenems and third generation cephalosporins – the best available antibiotics for treating multi-drug resistant bacteria.

The second and third tiers in the list – the high and medium priority categories – contain other increasingly drug-resistant bacteria that cause more common diseases such as gonorrhoea and food poisoning caused by salmonella.

G20 health experts will meet this week in Berlin. Mr Hermann Gröhe, Federal Minister of Health, Germany says “We need effective antibiotics for our health systems. We have to take joint action today for a healthier tomorrow. Therefore, we will discuss and bring the attention of the G20 to the fight against antimicrobial resistance. WHO’s first global priority pathogen list is an important new tool to secure and guide research and development related to new antibiotics.”

The list is intended to spur governments to put in place policies that incentivize basic science and advanced R&D by both publicly funded agencies and the private sector investing in new antibiotic discovery. It will provide guidance to new R&D initiatives such as the WHO/Drugs for Neglected Diseases initiative (DNDi) Global Antibiotic R&D Partnership that is engaging in not-for-profit development of new antibiotics.

Tuberculosis – whose resistance to traditional treatment has been growing in recent years – was not included in the list because it is targeted by other, dedicated programmes. Other bacteria that were not included, such as streptococcus A and B and chlamydia, have low levels of resistance to existing treatments and do not currently pose a significant public health threat.

The list was developed in collaboration with the Division of Infectious Diseases at the University of Tübingen, Germany, using a multi-criteria decision analysis technique vetted by a group of international experts. The criteria for selecting pathogens on the list were: how deadly the infections they cause are; whether their treatment requires long hospital stays; how frequently they are resistant to existing antibiotics when people in communities catch them; how easily they spread between animals, from animals to humans, and from person to person; whether they can be prevented (e.g. through good hygiene and vaccination); how many treatment options remain; and whether new antibiotics to treat them are already in the R&D pipeline.

“New antibiotics targeting this priority list of pathogens will help to reduce deaths due to resistant infections around the world,” says Prof Evelina Tacconelli, Head of the Division of Infectious Diseases at the University of Tübingen and a major contributor to the development of the list. “Waiting any longer will cause further public health problems and dramatically impact on patient care.”

While more R&D is vital, alone, it cannot solve the problem. To address resistance, there must also be better prevention of infections and appropriate use of existing antibiotics in humans and animals, as well as rational use of any new antibiotics that are developed in future.

**WHO priority pathogens list for R&D of new antibiotics**

**Priority 1: CRITICAL**

1. Acinetobacter baumannii, carbapenem-resistant
2. Pseudomonas aeruginosa, carbapenem-resistant
3. Enterobacteriaceae, carbapenem-resistant, ESBL-producing

**Priority 2: HIGH**

1. Enterococcus faecium, vancomycin-resistant
2. Staphylococcus aureus, methicillin-resistant, vancomycin-intermediate and resistant
3. Helicobacter pylori, clarithromycin-resistant
4. Campylobacter spp., fluoroquinolone-resistant
5. Salmonellae, fluoroquinolone-resistant
6. Neisseria gonorrhoeae, cephalosporin-resistant, fluoroquinolone-resistant

**Priority 3: MEDIUM**

1. Streptococcus pneumoniae, penicillin-non-susceptible
2. Haemophilus influenzae, ampicillin-resistant
3. Shigella spp., fluoroquinolone-resistant

Available from: http://www.who.int/mediacentre/news/releases/2017/bacteria-antibiotics-needed/en/

